# Effects of Tirzepatide on Body Composition, Metabolic Parameters, and Sleep Outcomes: A Real-World One-Year Prospective Study

**DOI:** 10.7759/cureus.107081

**Published:** 2026-04-15

**Authors:** Nikos Adamidis, Theodora Margariti, Vasiliki E Georgakopoulou, Sofia Adamidi, Athanasios Desalermos, Maria Kyventidou, Theodoros Koutrakos, Demosthenes E Bouros, Nektaria Papadopoulou, Sotirios Adamidis

**Affiliations:** 1 First Department of Internal Medicine, Sismanogleio Hospital, Athens, GRC; 2 Department of Endocrine Surgery, Helios University Hospital Wuppertal, Wuppertal, DEU; 3 Department of Pathophysiology, National and Kapodistrian University of Athens, Laiko General Hospital, Athens, GRC; 4 Department of Internal Medicine, Charlton Memorial Hospital, Fall River, USA; 5 Medical School, Harvard University, Cambridge, USA; 6 Department of Lifestyle Medicine, Athens Medical Group, Athens, GRC; 7 Department of Pulmonology, Athens Medical Group, Athens, GRC; 8 Department of Endocrinology, National and Kapodistrian University of Athens, Athens, GRC; 9 First Department of Internal Medicine, Athens Medical Group, Athens, GRC

**Keywords:** body composition, insulin resistance, obesity, sleep apnea, tirzepatide

## Abstract

Background

Tirzepatide, a dual glucose-dependent insulinotropic polypeptide (GIP) and glucagon-like peptide-1 (GLP-1) receptor agonist, has demonstrated substantial efficacy in reducing body weight and improving glycemic control in randomized trials; however, real-world data on its long-term effects remain limited. Our objective was to evaluate the one-year effects of tirzepatide on body composition, metabolic parameters, inflammation, and sleep-related outcomes in a real-world setting.

Methods

In this prospective study, 164 consecutive adult participants receiving tirzepatide in routine clinical practice were followed longitudinally. Anthropometric measurements, bioelectrical impedance-derived body composition indices, glycemic markers, inflammatory and biochemical parameters, and sleep-related indices were assessed at baseline and follow-up. Changes (D variables) were calculated as follow-up minus baseline values. Statistical analyses included descriptive statistics, paired comparisons, and correlation analyses.

Results

Tirzepatide treatment was associated with significant weight reduction (change in body weight (DWeight) -8.66 ± 8.02 kg), primarily driven by decreases in fat mass (change in fat mass (DFM) -11.34 ± 10.86 kg) and waist circumference (change in waist circumference (DWC) -10.11 ± 10.10), with relative preservation of lean mass (change in fat-free mass (DFFM) 0.87 ± 14.56 kg). Improvements were observed in glycemic control (change in glycated hemoglobin (DHbA1c) -0.59 ± 0.56%) and insulin resistance (change in homeostasis model assessment of insulin resistance (DHOMA-IR) -0.52 ± 0.29), along with reductions in inflammatory markers (change in C-reactive protein (DCRP) -1.48 ± 2.39 mg/L) and liver enzymes (change in aspartate aminotransferase (DAST) and alanine aminotransferase (DALT)). Sleep-disordered breathing improved, with a reduction in apnea-hypopnea index (change in apnea-hypopnea index (DAHI) -6.64 ± 6.63 events/h). Sex-specific differences were observed, with greater FM reduction in females and greater improvement in IR and sleep apnea indices in males.

Conclusions

In a real-world setting, tirzepatide demonstrates sustained benefits across adiposity, metabolic function, inflammation, and sleep outcomes over one year, with preservation of lean mass. These findings support its role as a comprehensive metabolic therapy.

## Introduction

Obesity and type 2 diabetes mellitus (T2DM) are chronic, relapsing, and closely interconnected metabolic disorders that contribute substantially to cardiovascular disease, chronic kidney disease, non-alcoholic fatty liver disease, reduced quality of life, and premature mortality. Because excess adiposity is a major driver of insulin resistance (IR) and cardiometabolic dysfunction, therapeutic strategies that achieve meaningful and sustained weight loss are increasingly viewed as central not only to obesity management but also to long-term diabetes prevention and metabolic risk reduction [1,2].

Tirzepatide is a once-weekly dual glucose-dependent insulinotropic polypeptide (GIP) and glucagon-like peptide-1 (GLP-1) receptor agonist that has emerged as one of the most effective pharmacologic options for weight reduction and glycemic improvement. In the SURPASS-2 trial, tirzepatide produced greater reductions in glycated hemoglobin (HbA1c) and body weight than semaglutide 1 mg in patients with T2DM, highlighting its strong metabolic efficacy in a high-risk population [3]. In parallel, the SURMOUNT-1 trial demonstrated that, in adults with obesity without diabetes, tirzepatide induced substantial and sustained weight loss over 72 weeks, with clinically important improvements in prespecified cardiometabolic measures [1]. Similar efficacy was also observed in adults with obesity and T2DM in the SURMOUNT-2 trial, supporting the consistency of its effect across different metabolic phenotypes [4].

As obesity is a chronic disease, however, the key clinical question is not only whether tirzepatide works initially but also whether its benefits persist over time and remain evident one year later or beyond. This issue is particularly relevant because long-term obesity treatment requires maintenance of weight reduction, metabolic improvement, and treatment tolerability. In the SURMOUNT-4 trial, continued tirzepatide therapy after an initial 36-week lead-in period maintained and further augmented weight loss over an additional 52 weeks, whereas treatment withdrawal was associated with substantial weight regain, emphasizing that ongoing therapy may be necessary for durable benefit [5]. Longer-term evidence has further strengthened this view: in participants with obesity and prediabetes from SURMOUNT-1, tirzepatide treatment over 176 weeks resulted in sustained weight reduction and a markedly lower risk of progression to T2DM than placebo [6]. Beyond weight and glycemic control, exploratory analyses from the SURPASS-4 trial also suggested favorable renal effects, including slower estimated glomerular filtration rate decline and reduced albuminuria, indicating that tirzepatide may exert broader organ-protective effects during longer follow-up [7].

Taken together, current evidence suggests that tirzepatide has the potential to modify the long-term trajectory of obesity-related metabolic disease rather than simply induce short-term weight loss. Nevertheless, evaluation of its effects at approximately one year and thereafter remains clinically important, since durability of response, maintenance after continued use, and the consequences of treatment interruption are all highly relevant to real-world decision-making. Therefore, an analysis focused on the long-term effects of tirzepatide one year later is warranted to better define the persistence of its benefits, its safety profile, and its implications for chronic obesity and diabetes management. We hypothesized that, in a real-world clinical setting, tirzepatide would lead to sustained improvements in body composition, characterized by preferential fat mass (FM) reduction with preservation of lean mass, along with concomitant improvements in glycemic control, systemic inflammation, and sleep-related outcomes over a one-year period.

Despite robust evidence from randomized controlled trials, real-world longitudinal data evaluating the durability of tirzepatide’s effects across multiple physiological domains remain limited, particularly over clinically relevant timeframes such as one year. Moreover, existing studies have largely focused on weight and glycemic outcomes, with less emphasis on detailed body composition, inflammatory status, and sleep-related parameters. Therefore, the present study aims to address these gaps by providing a comprehensive, real-world evaluation of tirzepatide’s multi-system effects over a one-year period.

The primary aim of this study was to evaluate the one-year effects of tirzepatide on body composition in a real-world setting. Secondary aims included assessment of metabolic, inflammatory, and hepatic parameters, while exploratory analyses examined sleep-related outcomes and behavioral factors.

## Materials and methods

Study design and setting

This prospective longitudinal study was conducted at the outpatient clinic setting of Athens Medical Group between December 1, 2024, and December 1, 2025. Participants were enrolled consecutively and followed from baseline to the predefined follow-up time point of approximately 12 months after treatment initiation. The study was designed to assess longitudinal changes in anthropometric, body-composition, metabolic, inflammatory, and sleep-related parameters under real-world clinical practice conditions.

Given the observational real-world design of the study, a formal a priori sample size calculation was not performed. Instead, the study aimed to include all consecutive eligible patients initiating tirzepatide during the study period to maximize external validity and reflect routine clinical practice. Nevertheless, the final sample size of 164 participants was considered adequate to detect clinically meaningful within-subject changes in primary outcomes (e.g., body weight and FM), based on effect sizes reported in previous randomized clinical trials of tirzepatide, where substantial reductions in body weight (≥5-10%) were consistently observed.

No formal sample size calculation was performed, as this was a real-world observational study based on consecutive patient enrollment. The Institutional Review Board of Athens Medical Group issued approval 106/28-11-2024.

Participant recruitment and follow-up procedures

Eligible participants were identified during routine outpatient visits and were invited to participate if they were considered appropriate candidates for tirzepatide treatment according to contemporary clinical practice and physician judgment. Potentially eligible individuals were informed about the aims of the study during their clinic visit and, where applicable, were contacted prospectively through scheduled outpatient follow-up, telephone communication, electronic messaging, or a combination of these methods to ensure attendance at reassessment visits and completion of follow-up measurements.

At enrollment, all participants underwent baseline clinical evaluation before initiation of tirzepatide. Follow-up assessments were scheduled prospectively at predefined intervals according to the clinical protocol of the center, with the principal analytic time point being approximately 12 months after treatment initiation.

Eligibility criteria

Adults aged ≥18 years who initiated tirzepatide therapy for obesity, excess adiposity with metabolic dysfunction, IR, prediabetes, T2DM, or related clinical indications were considered eligible for inclusion. Participants were required to have undergone baseline assessment before the first tirzepatide dose and to have at least one prospective follow-up visit with repeat clinical, anthropometric, biochemical, or body-composition measurements.

Exclusion criteria were pregnancy or lactation; active malignant disease; severe acute illness at enrollment; recent bariatric surgery; use of another anti-obesity injectable therapy during the observation period; inability to complete scheduled follow-up; or missing baseline data for the primary outcome.

Exposure to tirzepatide

The exposure of interest was treatment with tirzepatide administered as a once-weekly subcutaneous injection under routine clinical practice conditions, using the commercially available formulation (Mounjaro®, Eli Lilly and Company, Indianapolis, IN, USA), in accordance with standard clinical practice. All participants were newly initiated on tirzepatide therapy for the management of overweight or obesity and related metabolic risks. The starting dose was 2.5 mg or 5 mg once weekly, with dose escalation to 5 mg during the first month of treatment based on tolerability and clinical response, in accordance with standard prescribing practices.

Dose adjustments over time, including escalation, maintenance dose, temporary discontinuation, and treatment cessation, were recorded where applicable. Treatment was individualized according to patient response, adverse effects, and physician judgment.

In addition to pharmacological therapy, all participants received standardized lifestyle intervention as part of the clinical protocol. This included dietary counseling based on Mediterranean diet principles, emphasizing increased intake of vegetables, fruits, legumes, whole grains, and olive oil, moderate consumption of fish and poultry, and reduced intake of red meat, processed foods, and refined sugars. Participants were also encouraged to engage in regular physical activity, specifically at least 45 minutes of brisk walking daily for a minimum of five days per week.

Concomitant medications, excluding systemic corticosteroids and other agents known to significantly affect body weight or metabolic parameters, were continued and adjusted as clinically indicated during follow-up. All co-administered treatments were managed according to standard clinical care and were considered in the interpretation of study outcomes.

Adherence to lifestyle recommendations was encouraged through routine clinical follow-up and counseling; however, it was not formally quantified.

Baseline clinical assessment

At baseline, demographic and clinical information were recorded, including age, sex, indication for tirzepatide initiation, relevant medical history, comorbidities, and concurrent medications. A detailed physical examination and anthropometric assessment were performed before treatment initiation. Where applicable, participants were also evaluated for sleep-related breathing disorders, systemic inflammation, glycemic status, IR, liver function, and body-composition abnormalities.

Anthropometric measurements

Body weight was measured in light clothing and without shoes using a calibrated digital scale. Height was measured using a wall-mounted stadiometer at baseline, where available, and body mass index (BMI) was calculated as weight in kilograms divided by height in meters squared. The principal anthropometric endpoint in the available dataset was the change in body weight (DWeight), calculated as follow-up body weight minus baseline body weight.

Body-composition assessment

Body composition was assessed using bioelectrical impedance analysis (BIA) under standardized conditions, employing the BIA 101 device (Akern Srl, Florence, Italy), a validated non-invasive method for estimating body compartments. All measurements were conducted by trained personnel in accordance with the manufacturer’s instructions. To minimize physiological variability, participants were assessed under standardized conditions, including a fasting state, avoidance of vigorous physical activity prior to measurement, and abstinence from alcohol consumption for at least 24 hours. Measurements were performed with participants barefoot and without metallic objects.

The body-composition parameters evaluated included total body water (TBW), extracellular water (ECW), fat-free mass (FFM), body cell mass (BCM), and FM. As the BIA 101 device does not provide a direct measure of skeletal muscle mass, muscle tissue was included within the FFM compartment, which also comprises body water, bone, and organ tissue. In addition, waist circumference (WC) was measured as an index of central adiposity using a standardized protocol at the midpoint between the lowest rib and the iliac crest.

All body-composition variables were expressed as changes between baseline and follow-up, including DECW for ECW, DFFM for FFM, DBCM for BCM, DFM for FM, and DWC for WC. Change scores were calculated as follow-up minus baseline values; negative values indicated a reduction in the respective parameter over time, whereas positive values indicated an increase, depending on the physiological interpretation of each variable.

Glycemic and insulin-resistance assessment

Venous blood samples were collected at baseline and at follow-up after an overnight fast of at least eight to 12 hours. Glycemic control was evaluated using HbA1c, which reflects average blood glucose levels over the preceding two to three months. In the analysis, HbA1c is expressed as the change between follow-up and baseline values (DHbA1c), representing the longitudinal effect of tirzepatide on glycemic control.

Fasting plasma glucose and fasting insulin concentrations were measured, where available, using standard laboratory methods. IR was estimated using the homeostasis model assessment of IR (HOMA-IR), calculated according to the established formula: $HOMA-IR&quot;=(&quot;fasting insulin (&quot; &mu;&quot;U/mL)&quot; &times;&quot;fasting glucose (mg/dL)&quot; )/405$.

Changes in IR over time were expressed as the difference between follow-up and baseline HOMA-IR values (DHOMA-IR). Negative values indicated an improvement in insulin sensitivity, whereas positive values reflected worsening IR.

All biochemical analyses were performed in the same certified clinical laboratory using standardized assays and quality-control procedures to ensure consistency between baseline and follow-up measurements.

Inflammatory, biochemical, and alcohol consumption assessment

Fasting blood samples were collected at baseline and follow-up and analyzed for a range of inflammatory and biochemical markers. Systemic inflammation was assessed using C-reactive protein (CRP), while liver function was evaluated through measurement of aspartate aminotransferase (AST) and alanine aminotransferase (ALT). Serum amylase levels were also measured as part of routine biochemical monitoring during tirzepatide therapy, given their relevance for pancreatic safety. In the analysis, these parameters are expressed as longitudinal changes between baseline and follow-up, including DCRP for CRP, DAST for AST, DALT for ALT, and DAmylase for amylase.

Alcohol consumption was assessed using the Alcohol Use Disorders Identification Test (AUDIT), a validated screening tool for identifying hazardous and harmful alcohol use. The AUDIT score was calculated based on participants’ responses to the standardized questionnaire and was interpreted according to established cut-offs. Scores ranging from 0 to 7 were classified as low risk, indicating safe alcohol consumption with no need for intervention. Scores between 8 and 15 were considered indicative of hazardous drinking, for which brief advice on reducing alcohol intake was recommended. Scores of 16 to 19 reflected harmful drinking associated with an increased risk of adverse health outcomes, warranting closer monitoring and counseling. Scores of 20 or higher suggested a high likelihood of alcohol dependence, for which referral to a specialized addiction service was considered appropriate [8].

Changes in alcohol-related behavior over time were recorded in the analysis as DALCHOHOL, representing the difference in AUDIT score between follow-up and baseline. Negative values indicated a reduction in alcohol consumption risk, whereas positive values indicated worsening patterns of alcohol use.

All biochemical analyses were performed in the same certified clinical laboratory using standardized automated methods and internal quality control procedures to ensure consistency and reliability of measurements across time points.

Sleep-related assessment

Sleep-disordered breathing severity was assessed prospectively at baseline and follow-up using a standardized and validated diagnostic approach in accordance with the clinical protocol of the study center. Participants with clinical suspicion of sleep-disordered breathing, including symptoms such as snoring, daytime sleepiness, witnessed apneas, or obesity-related risk factors, were referred for sleep evaluation.

Assessment of sleep-disordered breathing was performed using attended overnight polysomnography, conducted according to established clinical guidelines. All recordings included continuous monitoring of respiratory parameters, such as airflow, respiratory effort, and oxygen saturation, with additional channels recorded depending on the diagnostic modality used. Sleep studies were analyzed by trained personnel using standardized scoring criteria consistent with current recommendations of the American Academy of Sleep Medicine (AASM) [9].

The apnea-hypopnea index (AHI) was used as the primary measure of sleep apnea severity and was defined as the total number of apneas and hypopneas per hour of sleep. Apnea was defined as a ≥90% reduction in airflow for at least 10 seconds, while hypopnea was defined as a ≥30% reduction in airflow lasting at least 10 seconds and associated with either oxygen desaturation or arousal, according to the scoring criteria applied.

AHI values were categorized, where applicable, into standard severity thresholds (mild: 5-14.9 events/hour, moderate: 15-29.9 events/hour, and severe: ≥30 events/hour) to facilitate clinical interpretation. In the dataset, AHI was expressed as a longitudinal change (DAHI), calculated as the difference between follow-up and baseline values. Negative values indicated an improvement in sleep-disordered breathing severity, whereas positive values reflected worsening of the condition.

All sleep assessments were performed using the same diagnostic modality and consistent scoring procedures at both time points to ensure comparability of measurements and minimize inter-measurement variability.

Data management

All data were recorded prospectively in a structured clinical database specifically designed for the study. Each participant was assigned a unique anonymized study identifier to ensure confidentiality, and all personally identifiable information was removed prior to statistical analysis. Data quality control procedures were implemented before analysis, including verification of data entry, identification of implausible or outlying values, and assessment of missing data. Given the prospective nature of the study and the variability in data availability across different endpoints, analyses were conducted on an available-case basis for each variable. Given the observational nature of the study, no formal adjustment for potential confounders was performed; however, concomitant treatments and lifestyle interventions were recorded and considered in the interpretation of the results. No formal sensitivity analyses were conducted; therefore, the potential impact of missing data on the robustness of the findings cannot be fully excluded.

Statistical analysis

Prior to inferential analysis, the distribution of continuous variables was assessed using graphical methods (histograms and Q-Q plots) and the Shapiro-Wilk test, where appropriate. Continuous variables are presented as mean ± standard deviation (SD). Categorical variables are presented as frequencies and percentages.

Changes over time were calculated as D variables (follow-up minus baseline). For normally distributed paired data, comparisons between baseline and follow-up values were performed using paired-samples t tests. Correlation analyses were conducted to evaluate associations between age and treatment-related changes using Pearson correlation coefficients.

A two-sided p-value <0.05 was considered statistically significant. Statistical analyses were performed using IBM SPSS Statistics for Windows, Version 26 (Released 2018; IBM Corp., Armonk, New York, United States).

## Results

A total of 164 participants, with a mean age of 47.62 (±12.54) years, were included in the study. Of these, 129 (78.7%) were female, and 35 (21.3%) were male. IR was highly prevalent, affecting 82.3% of participants, while hyperlipidemia (HLD) was present in 62.2% and hypertension (HTN) in 50.0% of the cohort. Liver disease was also common, reported in 56.1% of participants. T2DM was present in 22.0% of individuals, indicating that a substantial proportion of the population had established dysglycemia. Respiratory disease was observed in 13.4% of participants. In contrast, overt cardiovascular disease was less frequent. Coronary artery disease (CAD) was present in 7.3% of patients, myocardial infarction (MI) in 2.4%, and other cardiovascular diseases in 6.1%, while no cases of cerebrovascular disease (stroke) were recorded. Chronic kidney disease (CKD) and immunosuppression were relatively uncommon, affecting 4.9% and 1.8% of participants, respectively.

The mean baseline BW was 88.3 ± 22.7 kg. Body-composition analysis showed ECW of 19.0 ± 5.4 L and TBW of 49.5 ± 11.7 L. FFM was 64.2 ± 20.5 kg, while BCM was 39.0 ± 16.1 kg. FM was 31.6 ± 13.9 kg. The mean WC was 94.5 ± 20.6 cm, and the mean AHI was 14.0 ± 7.9 events/h.

Inflammatory and biochemical parameters included CRP at 5.9 ± 2.4 mg/L, AST at 45.1 ± 17.7 U/L (n = 93), ALT at 44.3 ± 18.4 U/L, and amylase at 43.0 ± 16.2 U/L. Alcohol consumption, assessed using AUDIT, had a mean score of 15.2 ± 7.3. Glycemic parameters included HbA1c at 6.37 ± 0.68% (n = 94) and HOMA-IR at 1.23 ± 0.49. Baseline descriptive statistics of the study population are presented in Table 1.

**Table 1:** Baseline characteristics of the study population (n = 164). AHI: apnea-hypopnea index; ALT: alanine aminotransferase; AST: aspartate aminotransferase; AUDIT: Alcohol Use Disorders Identification Test; BCM: body cell mass; BW: body weight; CAD: coronary artery disease; CKD: chronic kidney disease; CRP: C-reactive protein; CVD: cardiovascular disease; ECW: extracellular water; FFM: fat-free mass; FM: fat mass; HbA1c: glycated hemoglobin; HLD: hyperlipidemia; HOMA-IR: homeostasis model assessment of insulin resistance; HTN: hypertension; IR: insulin resistance; MI: myocardial infarction; TBW: total body water; T2DM: type 2 diabetes mellitus; WC: waist circumference

Variable	Value
Age (years)	47.62 ± 12.54
BW (kg)	88.3 ± 22.7
ECW (L)	19.0 ± 5.4
TBW (L)	49.5 ± 11.7
FFM (kg)	64.2 ± 20.5
BCM (kg)	39.0 ± 16.1
FM (kg)	31.6 ± 13.9
WC (cm)	94.5 ± 20.6
AHI (events/h)	14.0 ± 7.9
CRP (mg/L)	5.9 ± 2.4
AST (U/L)	45.1 ± 17.7
ALT (U/L)	44.3 ± 18.4
Amylase (U/L)	43.0 ± 16.2
AUDIT (score)	15.2 ± 7.3
HbA1c (%)	6.37 ± 0.68
HOMA-IR	1.23 ± 0.49
T2DM	36 (22.0%)
HTN	82 (50.0%)
CAD	12 (7.3%)
MI	4 (2.4%)
HLD	102 (62.2%)
CKD	8 (4.9%)
Respiratory disease	22 (13.4%)
Liver disease	92 (56.1%)
Immunosuppression	3 (1.8%)
Other CVD	10 (6.1%)
IR	135 (82.3%)

Changes in body composition following tirzepatide treatment

Treatment with tirzepatide was associated with substantial improvements in body composition and metabolic parameters. A marked reduction in body weight (DWeight -8.66 ± 8.02 kg) was observed, accompanied by a pronounced change in FM (DFM -11.34 ± 10.86 kg) and WC (DWC -10.11 ± 10.10), indicating a significant reduction in overall and central adiposity.

In contrast, changes in lean body compartments were minimal. FFM (DFFM 0.87 ± 14.56 kg) and body cell mass (DBCM 0.97 ± 14.06 kg) remained relatively stable, suggesting preservation of lean tissue during weight loss. Fluid-related parameters showed modest variation, with a slight increase in total body water (DTBW 2.21 ± 9.98 L) and minimal change in extracellular water (DECW 0.25 ± 5.27 L).

Improvements extended beyond body composition to include metabolic and clinical outcomes. Glycemic control improved, as reflected by reductions in HbA1c (DHbA1c -0.59 ± 0.56) and IR (DHOMA-IR -0.52 ± 0.29). Inflammatory burden was also reduced, with a decrease in CRP (DCRP -1.48 ± 2.39 mg/L).

Liver enzymes showed downward trends, with reductions in AST (DAST -5.00 ± 19.72 U/L) and ALT (DALT -6.52 ± 21.70 U/L), suggesting potential improvement in hepatic metabolic status. Amylase levels demonstrated a small decrease (DAmylase -3.82 ± 18.23 U/L), remaining within expected variability.

Notably, sleep-disordered breathing improved, with a reduction in AHI (DAHI -6.64 ± 6.63 events/h), indicating a clinically meaningful amelioration of sleep apnea severity. Additionally, alcohol consumption scores decreased (DAUDIT -5.08 ± 7.20), suggesting a concurrent improvement in behavioral risk factors. The changes in body composition following tirzepatide treatment are summarized in Table 2.

**Table 2:** Changes in body composition following tirzepatide treatment (n = 164). DWeight: change in body weight; DTBW: change in total body water; DECW: change in extracellular water; DFFM: change in fat-free mass; DBCM: change in body cell mass; DWC: change in waist circumference; DFM: change in fat mass; DAHI: change in apnea-hypopnea index; DCRP: change in C-reactive protein; DAST: change in aspartate aminotransferase; DALT: change in alanine aminotransferase; DAmylase: change in serum amylase; DAUDIT: change in Alcohol Use Disorders Identification Test; DHbA1c: change in glycated hemoglobin; DHOMA-IR: change in homeostasis model assessment of insulin resistance

Variable	Mean	SD
DWeight (kg)	-8.66	8.02
DTBW (L)	2.21	9.98
DECW (L)	0.25	5.27
DFFM (kg)	0.87	14.56
DBCM (kg)	0.97	14.06
DWC (cm)	-10.11	10.10
DFM (kg)	-11.34	10.86
DAHI (events/h)	-6.64	6.63
DCRP (mg/L)	-1.48	2.39
DAST (U/L)	-5.00	19.72
DALT (U/L)	-6.52	21.70
DAmylase (U/L)	-3.82	18.23
DAUDIT (score)	-5.08	7.20
DHbA1c (%)	-0.59	0.56
DHOMA-IR	-0.52	0.29

**Figure 1:**
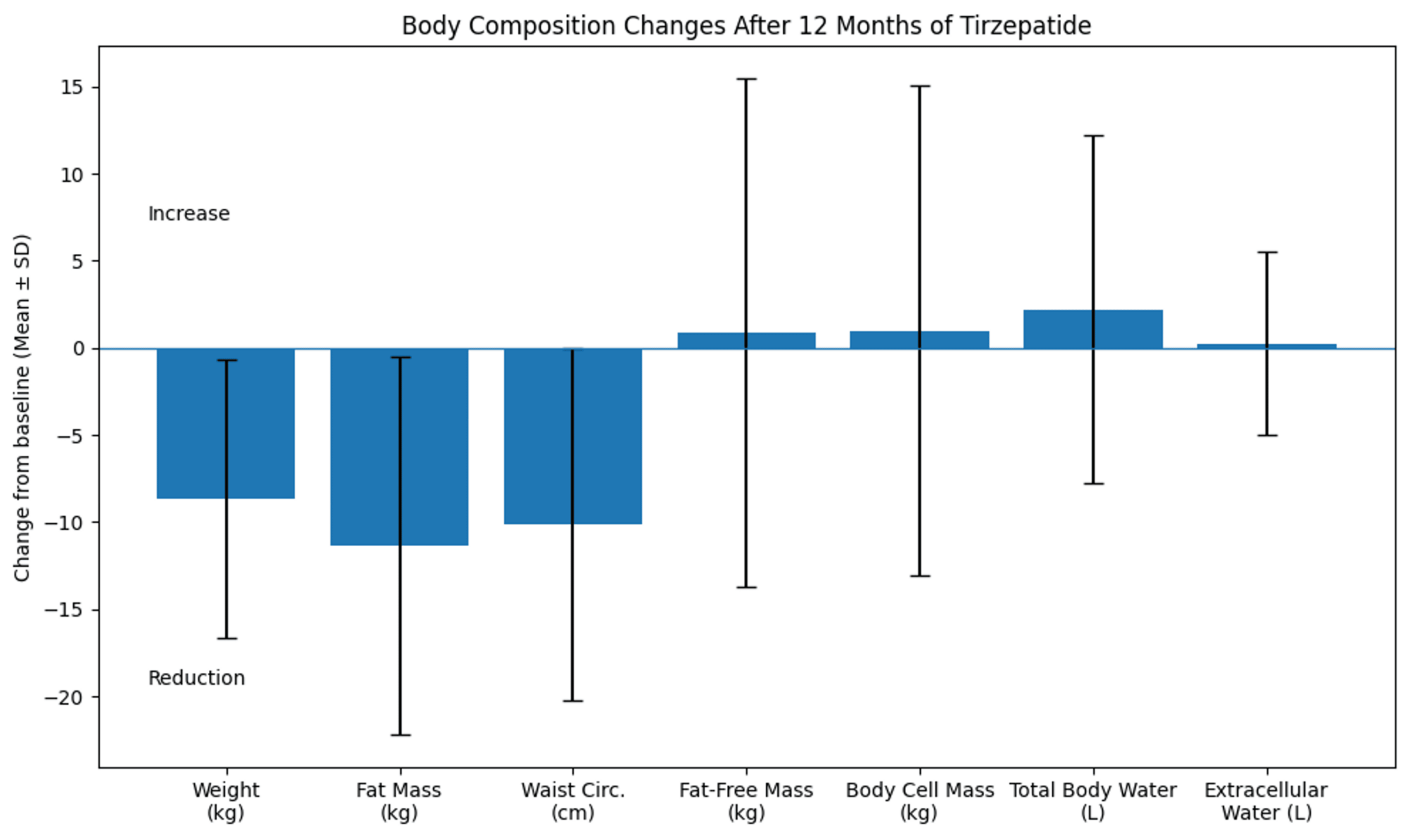
Changes in body composition parameters after 12 months of tirzepatide treatment. Values are presented as mean ± SD.

Figure 1 summarizes the changes in body composition parameters after 12 months of tirzepatide treatment.

Comparison of treatment-related changes between females and males

The comparison of treatment-related changes between females and males revealed several notable findings. Females demonstrated a greater increase in DFFM, whereas males exhibited a reduction (p = 0.002). Similarly, reduction in DFM was significantly greater in females compared to males (p = 0.001). Changes in DTBW also differed significantly between groups, with females showing an increase and males a decrease (p = 0.007). Regarding sleep-related outcomes, males exhibited a greater reduction in DAHI compared to females (p = 0.032). In addition, a significant difference was observed in DHOMA-IR, with males showing a greater reduction compared to females (p = 0.015). In contrast, no statistically significant differences between sexes were observed in DWeight, DECW, DBCM, DWC, DCRP, DAST, DALT, DAmylase, DAUDIT, or DHbA1c (all p > 0.05). Table 3 summarizes the comparisons of changes by gender.

**Table 3:** Comparison of treatment-related changes between females (n = 129) and males (n = 35). DWeight: change in body weight; DTBW: change in total body water; DECW: change in extracellular water; DFFM: change in fat-free mass; DBCM: change in body cell mass; DWC: change in waist circumference (cm); DFM: change in fat mass; DAHI: change in apnea-hypopnea index; DCRP: change in C-reactive protein; DAST: change in aspartate aminotransferase; DALT: change in alanine aminotransferase; DAmylase: change in serum amylase; DAUDIT: change in Alcohol Use Disorders Identification Test; DHbA1c: change in glycated hemoglobin; DHOMA-IR: change in homeostasis model assessment of insulin resistance Statistical test: independent samples t-test

Variable	Female (Mean ± SD)	Male (Mean ± SD)	t-value	p-value
DWeight (kg)	-9.08 ± 8.18	-7.25 ± 7.38	-1.13	0.258
DTBW (L)	3.13 ± 10.67	-1.61 ± 4.92	2.74	0.007
DECW (L)	0.24 ± 5.41	0.31 ± 4.92	-0.05	0.961
DFFM (kg)	3.22 ± 9.85	-8.28 ± 24.07	3.16	0.002
DBCM (kg)	1.26 ± 15.51	-0.10 ± 6.42	0.36	0.718
DWC (cm)	-10.29 ± 9.54	-9.50 ± 11.96	-0.39	0.697
DFM (kg)	-13.84 ± 11.79	-5.43 ± 4.70	-3.36	0.001
DAHI (events/h)	-6.28 ± 6.36	-12.80 ± 8.90	2.17	0.032
DCRP (mg/L)	-1.44 ± 2.42	-2.16 ± 1.99	0.65	0.515
DAST (U/L)	-4.95 ± 19.46	-5.76 ± 26.54	0.09	0.930
DALT (U/L)	-6.74 ± 22.07	-2.60 ± 14.77	-0.41	0.681
DAmylase (U/L)	-3.82 ± 18.61	-3.96 ± 10.48	0.02	0.987
DAUDIT (score)	-5.13 ± 7.24	-4.20 ± 7.26	-0.28	0.781
DHbA1c (%)	-0.59 ± 0.57	-0.58 ± 0.38	-0.04	0.970
DHOMA-IR	-0.49 ± 0.28	-0.90 ± 0.10	2.47	0.015

Correlation of age with treatment-related changes

Age was negatively correlated with DHbA1c (r = -0.290, p = 0.005) and DAHI (r = -0.236, p = 0.025). No significant correlations were observed between age and changes in body composition parameters, including DFM, DFFM, DBCM, DTBW, DECW, DWC, or DWeight (all p > 0.05). Similarly, age was not significantly associated with changes in inflammatory or biochemical markers, including DCRP, DAST, DALT, Damylase, or DAUDIT (all p > 0.05), nor with DHOMA-IR. Table 4 summarizes the correlation of age with treatment-related changes.

**Table 4:** Correlation of age with treatment-related changes. DAHI: change in apnea-hypopnea index; DALT: change in alanine aminotransferase; DAmylase: change in serum amylase; DAST: change in aspartate aminotransferase; DBCM: change in body cell mass; DCRP: change in C-reactive protein; DECW: change in extracellular water; DFFM: change in fat-free mass; DFM: change in fat mass; DHbA1c: change in glycated hemoglobin; DHOMA-IR: change in homeostasis model assessment of insulin resistance; DTBW: change in total body water; DWeight: change in body weight; DWC: change in waist circumference; DAUDIT: change in Alcohol Use Disorders Identification Test Statistical test: Pearson correlation coefficient (two-tailed)

Variable	r	p-value
DHbA1c (%)	-0.290	0.005
DAHI (events/h)	-0.236	0.025
DFM (kg)	0.046	0.759
DWC	0.035	0.680
DBCM (kg)	-0.102	0.352
DFFM (kg)	0.060	0.566
DECW (L)	-0.159	0.152
DTBW (L)	-0.046	0.659
DWeight (kg)	0.077	0.367
DCRP (mg/L)	-0.050	0.629
DAST (U/L)	-0.090	0.392
DALT (U/L)	-0.035	0.740
DAmylase (U/L)	0.032	0.761
DAUDIT	-0.071	0.502
DHOMA-IR	-0.114	0.465

**Figure 2:**
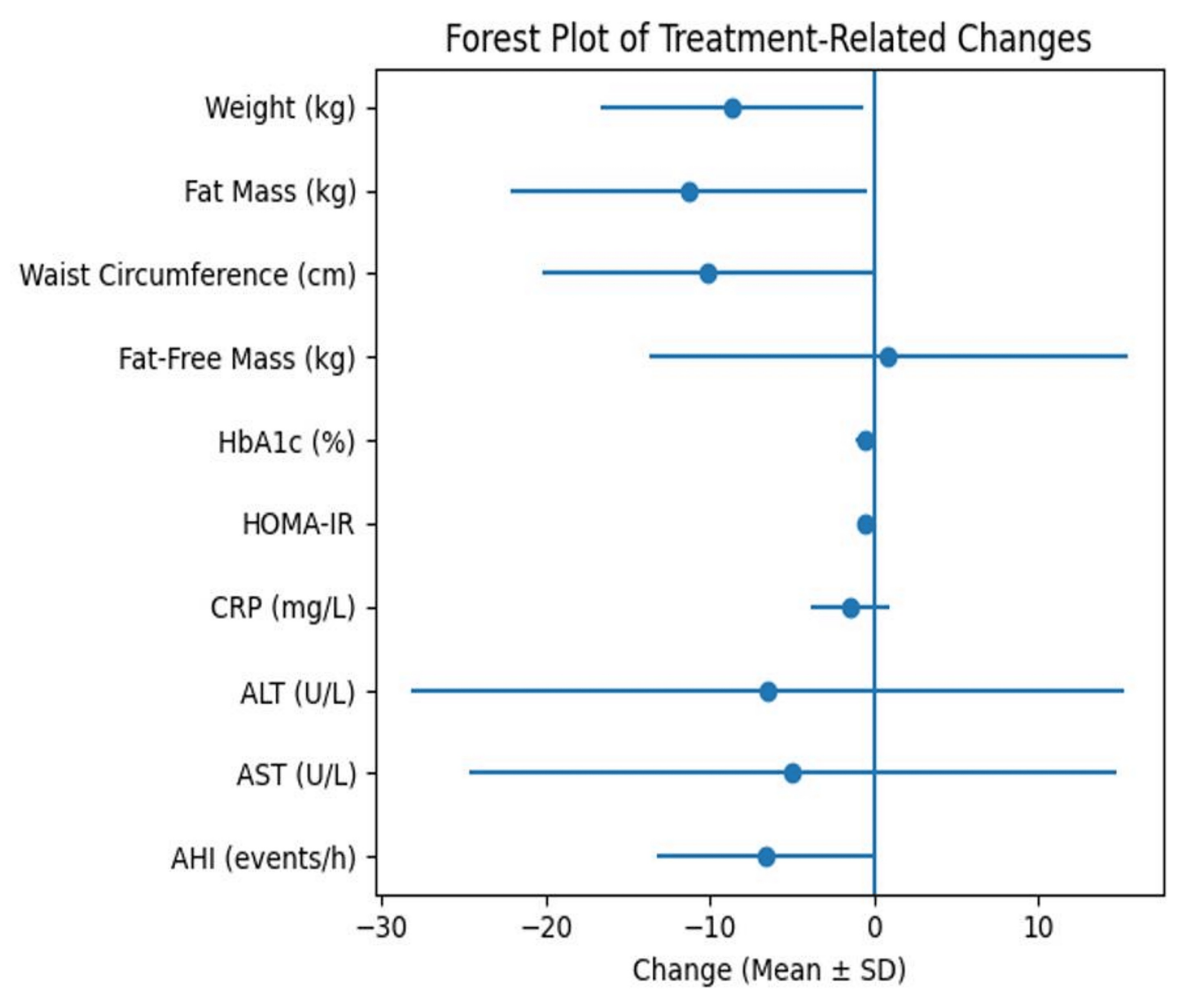
Changes in anthropometric, metabolic, inflammatory, and sleep-related parameters after 12 months of tirzepatide treatment. HbA1c: glycated hemoglobin; HOMA-IR: homeostasis model assessment of insulin resistance; CRP: C-reactive protein; ALT: alanine aminotransferase; AST: aspartate aminotransferase; AHI: apnea-hypopnea index

Figure 2 summarizes the changes in anthropometric, metabolic, inflammatory, and sleep-related parameters after 12 months of tirzepatide treatment.

## Discussion

In this prospective real-world study, tirzepatide treatment over approximately one year resulted in consistent improvements across adiposity, metabolic control, inflammation, and sleep-related outcomes. Importantly, these findings not only confirm the efficacy observed in randomized trials but also demonstrate that these effects are largely preserved under real-world conditions, where treatment intensity and adherence are less controlled.

The magnitude of weight reduction, although lower than that reported in the SURMOUNT-1 and SURPASS-2 trials [1,10], should be interpreted in the context of routine clinical practice. Rather than representing reduced efficacy, this difference likely reflects heterogeneity in dosing, adherence, and patient characteristics. Notably, the qualitative pattern of weight loss is particularly informative: the predominant reduction in DFM, with relative stability of DFFM and DBCM, suggests a selective depletion of adipose tissue. This is a critical distinction, as preservation of lean mass is associated with improved metabolic adaptation and reduced risk of weight regain, supporting the concept that tirzepatide induces a metabolically favorable weight-loss phenotype [11].

The concurrent improvements in DHbA1c and DHOMA-IR reinforce the central role of dual incretin signaling in modulating glucose homeostasis. While part of this effect is weight-mediated, the magnitude and consistency of improvement suggest additional weight-independent mechanisms, including enhanced insulin sensitivity and modulation of adipose tissue function [12]. This is further supported by the observed reduction in DCRP, indicating attenuation of chronic low-grade inflammation, a key driver of IR. Similarly, reductions in DAST and DALT point toward improved hepatic metabolic status, likely reflecting decreased hepatic fat accumulation and inflammatory activity [13,14].

The improvement in DAHI provides additional insight into the systemic impact of tirzepatide. Although weight loss remains the primary determinant of sleep apnea improvement, the extent of reduction suggests that metabolic and inflammatory changes may also contribute. This aligns with emerging evidence that incretin-based therapies may influence respiratory physiology beyond simple mechanical effects of weight loss [15].

The observed sex-specific differences highlight the heterogeneity of response. The greater reduction in DFM in females, contrasted with larger improvements in DAHI and DHOMA-IR in males, suggests differential partitioning of metabolic adaptation. These findings are consistent with known sex differences in fat distribution and insulin sensitivity, where women tend to exhibit greater subcutaneous fat loss and men greater visceral and metabolic improvement [16]. Such differences may have implications for individualized treatment strategies.

The inverse association between age and both DHbA1c and DAHI is notable and somewhat counterintuitive. While aging is generally associated with reduced metabolic plasticity, the greater improvements observed in older individuals may reflect higher baseline disease burden or differences in treatment adherence and responsiveness. The absence of association between age and body composition changes suggests that the weight-reducing effect of tirzepatide is robust across age groups.

A reduction in AUDIT score was also observed; however, this finding should be interpreted with caution, as no alcohol-specific intervention was implemented and the AUDIT is a self-reported measure prone to reporting bias. Although emerging evidence suggests that incretin-based therapies may influence reward-related behaviors [17-19], the present study was not designed to evaluate this effect, and the finding should be considered exploratory.

From a clinical perspective, these findings support the role of tirzepatide as a multi-system metabolic therapy rather than a simple weight-loss agent. The combination of adipose tissue reduction, preservation of lean mass, improvement in IR, and reduction in inflammation suggests a broader disease-modifying potential.

This study has several strengths. Its prospective design and real-world setting enhance the generalizability of the findings beyond controlled trial environments. The comprehensive assessment of outcomes, including detailed body composition, metabolic, inflammatory, and sleep-related parameters, provides a multidimensional evaluation of treatment effects. Additionally, the analysis of sex-specific and age-related differences offers further insight into variability in treatment response, supporting more personalized approaches to therapy.

However, these results should be interpreted in light of certain limitations. First, the absence of a control group limits causal inference, as the observed improvements cannot be attributed exclusively to tirzepatide and may partly reflect concurrent lifestyle interventions or regression to the mean. In this context, although lifestyle modification was standardized as part of routine care, its independent contribution was not quantitatively assessed and therefore cannot be fully separated from the pharmacological effect. Second, the variability in sample size across outcomes and the use of available-case analysis introduce potential bias related to missing data. Third, body composition was assessed using BIA, which, while practical in real-world settings, may not capture regional fat distribution with the same precision as gold-standard methods such as dual-energy X-ray absorptiometry or magnetic resonance imaging. In addition, the absence of a formal sample size calculation, lack of standardized assessment of adherence to lifestyle interventions, and variability in follow-up intervals reflect the real-world design of the study but may limit strict reproducibility. Finally, the predominance of female participants may limit the generalizability of the findings to male populations.

## Conclusions

Tirzepatide treatment over one year is associated with clinically meaningful improvements across multiple domains, including adiposity, glycemic control, inflammation, and sleep-disordered breathing. Importantly, weight loss is predominantly driven by reductions in FM with preservation of lean tissue, supporting a metabolically favorable profile. These findings support the potential role of tirzepatide as a multifaceted metabolic therapy in real-world clinical practice, although the observed effects should be interpreted in the context of the study’s observational design and cannot be considered definitive evidence of causality.
